# A rural ambulance service in Northern Togo improves access to emergency care for women with obstetric complications

**DOI:** 10.7189/jogh.14.04201

**Published:** 2024-11-08

**Authors:** Margaret Fagan, Samantha Levano, Jessica Haughton, Komivi Badohoun, Désiré Dabla, Assiongbonvi Kangni-Zovoin, Messan Midokpor, Wiyao Katchoou, Ekla Agba, Kevin P Fiori

**Affiliations:** 1 Department of Pediatrics, Albert Einstein College of Medicine, Bronx, New York, USA; 2 Community Health Systems Lab, Integrate Health/Santé Intégrée, Kara, Togo; 3 Department of Family & Social Medicine, Albert Einstein College of Medicine, Bronx, New York, USA; 4 Ministry of Health, Public Hygiene, and Universal Access to Health Care, Kara, Togo

## Abstract

**Background:**

Maternal mortality remains high in sub-Saharan Africa, with little progress made in the last 20 years. The provision of emergency obstetric care has been shown to have the greatest effect in reducing maternal mortality in countries with high maternal mortality ratios, especially when paired with an emergency transport service. Integrate Health has partnered with the Togolese Ministry of Health to improve maternal and child health via the integration of a free ambulance service into a pre-existing primary care model. In this study, we aim to describe the implementation of this service and assess its effectiveness on access to emergency obstetric care by estimating its coverage of women with obstetric complications.

**Methods:**

This is a retrospective cross-sectional study using routinely collected data from ambulance logbooks. The study was restricted to pregnant or postpartum woman in four districts of Northern Togo. For each patient transported, the variables collected included date of transport, destination, patient information, kilometres travelled, and reason for transport. Complicated obstetric cases were defined by reason for transport and included maternal haemorrhage, complicated birth, and signs of danger. Estimated coverage of major obstetric complications was calculated using population estimates per fiscal year, the birth rate (3.7%) in Togo, and the assumption that 15% of pregnant women will have a complication.

**Results:**

Between July 2020 and June 2023, there were 2926 maternal patients transported by the ambulance service. Of these, 1030 were reported as complicated obstetric cases. Estimated coverage of obstetric complications increased over time and as the programme expanded, from 18% in 2020 to 35.7% in 2021, and 66.5% in 2022.

**Conclusions:**

Our findings demonstrate that implementing a rural ambulance service in a region with historically high maternal mortality rates may improve maternal access to emergency obstetric care. The success of our ambulance service was likely due to the fact that it is free, available 24/7, easily accessible, operated by trained staff and community members, and integrated into a pre-existing primary care programme with well-resourced health care centres.

Maternal mortality ratios (MMR) in sub-Saharan Africa remain the highest in the world, despite vast improvements made in other regions [1]. In 2020, the World Health Organization (WHO) developed Sustainable Developmental Goal (SDG) Target 3.1 to reduce the global MMR to less than 70 deaths per 100 000 live births in the next 10 years [2]. Togo, a small country in West Africa, will likely fall short of this SDG with an MMR of 399 deaths per 100 000 live births in 2020 and little progress made since 2000 [3].

It is often challenging to estimate MMRs because maternal mortality is a relatively rare event and is poorly reported, with less than 40% of countries currently having a complete civil registration system in place to record maternal deaths [4]. Importantly, Togo is one of the countries that does not have such a system. To circumvent this problem, the WHO and the United Nations (UN) created six process indicators that can be used instead to monitor maternal mortality progress in a region or country [5]. These indicators include:

1) availability of emergency obstetric care (EmOC) services

2) geographical distribution of EmOC

3) proportion of all births in EmOC facilities

4) met need for EmOC (proportion of women with major obstetric complications who receive care)

5) caesarean section as a proportion of all births (caesarean section rate (CSR))

6) facility direct obstetric case fatality rate.

The provision of EmOC has been shown to have the greatest effect in reducing maternal mortality in countries with high MMRs [6]. Providing complementary emergency transport is a necessary component of providing emergency care in low resource settings [7]. In fact, evidence suggests that emergency obstetric interventions were significantly more effective when paired with an emergency transport service, which demonstrates the potential use of these interventions to achieve SDG 3.1 in sub-Saharan Africa [8]. Recent studies on emergency transport services in Burundi [9], Uganda [10], India [11], South Sudan [12], and Tanzania [13] have used the WHO/UN process indicators, primarily met need for EmOC and the CSR, to evaluate how their service influences maternal and child health (MCH). Despite their value, emergency transport services are not widely utilised in sub-Saharan Africa, due to long travel distances, difficult terrain, and maintenance costs [14]. The services that do exist are difficult to evaluate due to limited data collection and resources.

Togo is a francophone country in western sub-Saharan Africa with a population of almost nine million people, historically poor access to health care, and persistently high morbidity and mortality. Only 30% of the population uses public facilities, one in 15 children die before their fifth birthday, and the maternal mortality rate is 14 times higher than in high-income countries [15–17]. There are only four physicians per 100 000 people with resources concentrated in the few urban areas, which leaves the predominately rural communities without access to care. In 2010, the Togolese government partnered with other health organisations to address these disparities and achieve universal health coverage. Integrate Health (IH), an international non-governmental organisation (NGO), was identified as one such partner organisation and has since implemented the Integrated Primary Care Program (IPCP) to expand access to care across five districts in the Kara region of northern Togo. The IPCP uses a community engagement approach to implement evidence-based interventions designed to improve MCH, including subsidised service fees, case management using community health workers, clinical mentoring by peer coaches, and basic infrastructure and equipment improvements [18]. In 2015, IH integrated a free rural ambulance service into the pre-existing IPCP, which was designed to improve urgent access to quality health care for pregnant and postpartum women and children under five years old. Integrate Health has implemented the IPCP and ambulance referral service with the goal to both expand health care and provide evidence on the effectiveness of such interventions to the Togolese Ministry of Health with the hope that they can be transformed into national policies through government funding and support. Evidence supporting these interventions can also demonstrate the potential use of the IPCP and ambulance referral models in neighbouring countries with similar MCH needs targeted for IH expansion.

In this study, we aim to describe the implementation of the IH rural ambulance service in northern Togo and assess the effectiveness of the rural ambulance service in expanding access to EmOC by estimating the met need through the coverage of women with obstetric complications.

## METHODS

### Ambulance intervention

This was a retrospective cross-sectional study utilising data routinely collected by the IH rural ambulance service. This service consists of a motorised tricycle with an attached covered trailer, equipped to transport the driver, one patient, and one accompanying passenger. Ambulance use was restricted to pregnant and postpartum women who needed urgent transport for health centre delivery with or without obstetric complications and children 0–5 years old who were suffering from a serious illness or injury.

There are other public ambulances active in the Kara region, which are stationed in the city centre of each district. Most of the region is comprised of dirt roads that can be challenging, if not impossible, to navigate by car, especially during the rainy season from May to November. These other ambulances operate by car and, therefore, experience significant challenges accessing community members in need. Additionally, these ambulances charge passengers by distance travelled, meaning that rural communities pay the most for its use. For these reasons, patients and their families are often forced to seek alternative solutions and transport options. The IH rural ambulance service was implemented with these barriers in mind to improve access to care for rural communities in the Kara region.

### Study setting

**Figure 1:**
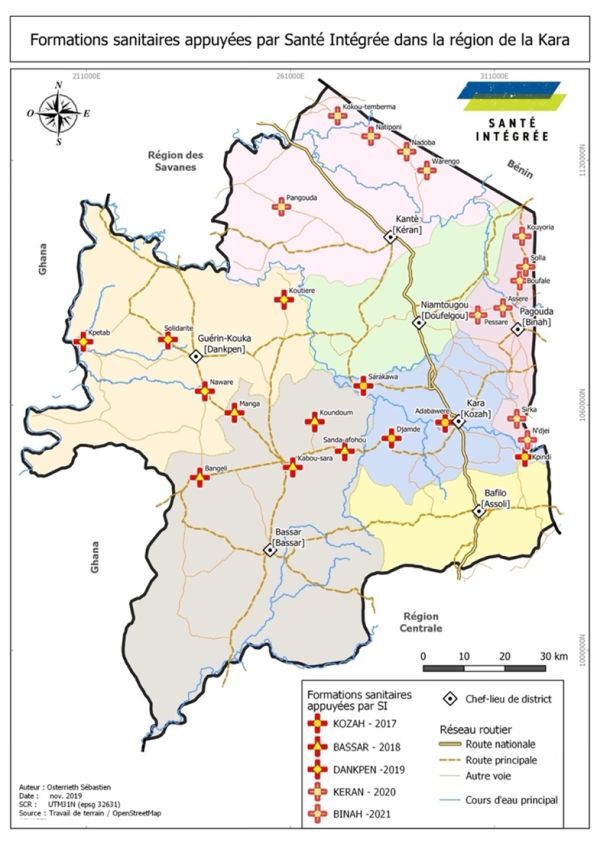
Integrate Health supported Village Health Centres in the Kara region. Figure was developed by Sébastien Osterrieth, who granted permission for its use and publication.

The IH rural ambulance service was made available in five districts in the Kara region of northern Togo, with an estimated population of 834 351 (**Figure 1**) [19]. Ambulance services were available to eligible pregnant and postpartum women and children aged zero to five years in the catchment populations of selected districts: Kozah, Bassar, Keran, Dankpen, and Binah (**Table 1**). Most of the districts are rural except for Kozah, which includes the capital city of Kara and is semi-urban.

**Table 1:** Descriptive statistics of districts served by Integrate Health Ambulance Referral Service in Northern Togo, July 2020 – June 2023

Variable (%)	Kozah* (10.4%)	Bassar (24.5%)	Dankpen (27.6%)	Keran† (22.8%)	Binah‡ (14.6%)	Total (100.0%)
Estimated catchment population (100.0)	50 562	118 772	133 491	110 379	70 665	483 869
Estimated pregnant women (3.7)	1871	4395	4939	4084	2615	17 904
Estimated pregnant women with complications (0.6§; 15.0ǁ)	281	659	741	613	392	2686

*Integrate Health Ambulance Referral Service not available in Adabawere, which was excluded in estimates.

†Integrate Health Ambulance Referral Service not available until September 2020.

‡Integrate Health Ambulance Referral Service not available until July 2021.

§Percent of estimated catchment population.

ǁPercent of estimated pregnant women.

There were 25 total IH-supported village health centres (VHC) across the five districts, one hospital in the centre of each of the five districts, and one university hospital in the capital city of Kara. Most VHCs were equipped with at least one nurse, birth attendant, and pharmacy manager, however, this varied slightly by catchment area. Services offered at the VHC included uncomplicated childbirth assistance, family planning services, prenatal and postpartum care, and the prevention and treatment of transmissible diseases, such as sexually transmitted infections (STIs), HIV, malaria, and other common childhood illnesses. Complicated pregnancies and serious or complicated illnesses were referred to the nearest district hospital. VHCs partially meet the WHO/UN set requirements of a Basic EmOC facility and the district hospitals meet all requirements of a Comprehensive EmOC facility [5].

There was one IH ambulance stationed at each VHC where it was maintained 24/7 by two ambulance drivers with alternating shifts. Ambulance drivers were nominated by their community and then trained by IH in basic medical care, navigation, and protocols for ambulance maintenance and data recording. To ensure timely communication with VHCs, ambulance drivers were equipped with a cell phone with prepaid credits included.

The need for an ambulance and subsequent dispatch was identified via set protocols, acute clinical assessments, and communication between the VHC head nurse, community health workers (CHWs), and ambulance drivers. There are three ways in which the ambulance can be requested and dispatched. First, CHWs could notify the VHC if they determined an urgent ambulance need when routinely monitoring women and children in the districts. The CHWs assessed women for ambulance need due to obstetric complications using the standardised definitions for complications (i.e. woman having haemorrhage, signs of danger, or complicated birth) described in the ambulance protocol. The head nurse or the care provider at the VHC would then dispatch the ambulance to the community member’s location and the driver would transport the patient back to the VHC, where they will either stay or be further transported to a district hospital, depending on the severity of their condition. Community members could also bypass the CHWs and call the VHC or the ambulance driver directly to explain their need for transport. If the ambulance driver is called directly, they must contact the VHC before going to the patient’s location. In addition to CHWs, ambulance drivers were also trained to assess women for obstetric complications in the community. Finally, women already at the VHC could also be transported by the ambulance if in need of more intensive care. The health care team at the VHC would determine the ambulance need by assessing women already at the VHC for obstetric complications defined in the protocol and risk stratifying women for additional clinical risk factors such as age, hypertension, proteinuria, and risk of malaria. If a woman had an obstetric complication or more than two risk factors, then health care team would transfer her to the district hospital via the ambulance. In all cases, the head nurse at the VHC made the final decision regarding ambulance dispatch, using both the described protocol and completing a clinical assessment of the patient depending on the acuity of the case.

### Data collection

Data was collected in an ambulance logbook by drivers and transcribed into an electronic database by data entry operators. For each patient transferred, the ambulance driver recorded data on a standardised transport sheet. Pertinent variables included: name, age, and sex of the patient, date of transport, ambulance site, destination of transport, reason for transport, and kilometres travelled. Reasons for transport for maternal patients included: haemorrhage, signs of danger, active labour, complicated birth, and other. Ambulance drivers may report more than one reason for transport of the patient.

### Study population

This study was limited to women transferred by the rural ambulance service who were at risk or suffering from obstetric complications in the Bassar, Dankpen, and Keran districts between July 2020 and June 2023 and in the Binah district between July 2021 and June 2023. The staggered launch of the ambulance service in Binah corresponds with the launch of the IPCP initiative in the district in 2021. Patients transported in the Kozah district were excluded due to inflated coverage of the women with obstetric complications (225.2% of expected cases), likely due to neighbouring communities migrating into the district for services.

Complicated obstetric cases were defined as any woman transported for haemorrhage, signs of danger, or complicated birth (**Box 1**). These definitions were selected according to the standardised WHO/UN definitions (e.g. haemorrhage, complicated birth) and those utilised by the IH IPCP programme team (e.g. signs of danger) [5]. The VHCs in Togo regularly utilise the signs of danger checklist as part of a robust protocol. Signs of danger include several risk factors and encompass other EmOC categories identified by the WHO/UN. If a woman had three or more signs of danger, the trained health officials would deem the case complicated enough to refer the patient immediately to a district hospital (i.e. comprehensive EmOC facility).

Box 1 **Definitions of complicated obstetric cases by Integrate Health Ambulance Referral Service**

1. Haemorrhage: bleeding during or after pregnancy

2. Signs of danger (three or more)

a. general medical pathologies: severe anaemia, malaria, HIV+, infection (signified by fever, vomiting, dehydration), malnutrition

b. genital bleeding

c. hypertension/albuminuria/oedema

d. multiple pregnancy

e. young mother <18 or small/narrow pelvis

f. history of complicated birth: stillbirth, prematurity, spontaneous abortions, pre-eclampsia

3. Complicated birth

a. eclampsia/preeclampsia/convulsions

b. cephalopelvic disproportion/scarred uterus

c. malpresentation of foetus

d. prolonged labour: lack of effort, stationary labour, dystocia

e. premature rupture of membranes

### Study measures and analysis

To estimate the effectiveness of the IH rural ambulance service in expanding access to EmOC in northern Togo, we compared the actual number of women with complicated obstetric cases who received care from the IH rural ambulance service with the expected number of complicated obstetric cases in the region, in accordance with the WHO/UN process indicator for met need for EmOC. We sourced the actual number of cases from the data collected in and transcribed from the ambulance logbook. We estimated the expected number of complicated obstetric cases in the region using population-level data routinely reported by VHCs in the District Health Information System (DHIS2), which is an open source, web-based health management information system used in 73 low- and middle-income countries, including Togo, to collect and process health facility data [20]. Population-level measures reported in DHIS2, including estimates of the catchment population by district and year and the estimated birth rate in Togo (3.7%) across the study period, were sourced from Togo’s Institut National de la Statistique et des Etudes Economiques et Démographiques. We utilised the catchment population estimates and birth rate reported in DHIS2 to calculate the expected number of pregnant women. We then referenced the WHO estimate that 15% of pregnant women will develop a potentially life-threatening complication that requires skilled care to estimate the number of pregnant women with complicated obstetric cases in the region [5]. The coverage of complicated obstetric cases was reported as a rate (number of actual complicated obstetric cases reported by IH rural ambulance service/estimated number of complicated obstetric cases).

## RESULTS

There were 2926 pregnant or postpartum women transported by the IH rural ambulance service between July 2020 and June 2023. Of these women, 734 (25.1%) were transported for labour, 446 (15.2%) for signs of danger, 430 (14.7%) for complicated birth, 199 (6.8%) for haemorrhage, 1031 (35.2%) for other reasons, and 216 (7.4%) for reasons not reported (**Table 2**). Approximately 576 (19.7%) of women were transported to the VHC, compared to 2302 (78.7%) transported to the district hospital or 48 (1.6%) transported to both. The average distance travelled to a VHC was 13.7 km while the average distance travelled to a hospital was 50.1 km.

**Table 2:** Summary of pregnant and postpartum transports by Integrate Health Ambulance Referral Service in four districts in northern Togo, July 2020 – June 2023

Variables	Bassar		Dankpen		Keran*		Binah†		Total
**Total women transported, n (%)**	598 (20.4)		605 (20.7)		940 (32.1)		783 (26.8)		2926 (100.0)
**Reasons for transport, n (%)**									
Labour	112 (18.7)		185 (30.6)		189 (20.1)		248 (31.7)		734 (25.1)
Signs of danger	98 (16.4)		57 (9.4)		133 (14.1)		158 (20.2)		446 (15.2)
Complicated birth	114 (19.1)		108 (17.9)		141 (15.0)		67 (8.6)		430 (14.7)
Haemorrhage	39 (6.5)		52 (8.6)		60 (6.4)		48 (6.1)		199 (6.8)
Other reason	154 (25.8)		194 (32.1)		398 (42.3)		285 (36.4)		1031 (35.2)
Reason not reported	93 (15.6)		54 (8.9)		42 (4.5)		27 (3.4)		216 (7.4)
**Distance travelled per woman transported, km**									
Average distanced travelled	60.1		20.9		47.6		42.0		43.2
Average distanced travelled to Hospital	65.8		20.6		68.7		46.1		50.1
Average distanced travelled to Village Health Centre	23.7		23.9		10.4		12.4		13.7

*Integrate Health Ambulance Referral Service not available until September 2020.

†Integrate Health Ambulance Referral Service not available until July 2021.

There were 1030 (35.2%) women who met the criteria for complicated obstetric cases. Of these women, 107 (10.4%) were transported to the VHC, 896 (87.0%) to the district hospital, and 27 (2.6%) to both. The average distance travelled to a VHC was 12.0 km while the average distance travelled to a hospital was 51.7 km. There are no available socio-demographic characteristics to compare women with and without complicated obstetric cases.

**Figure 2:**
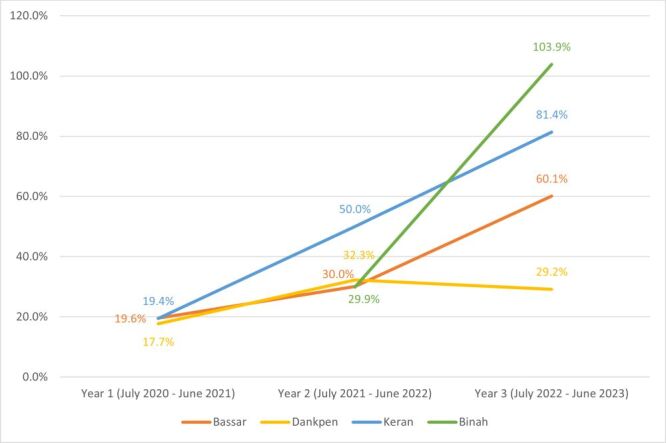
Estimated coverage of complicated obstetric cases by Integrate Health Ambulance Referral Service in Northern Togo by District, July 2020 – June 2023.

Between July 2020 and June 2021, there were 117 complicated obstetric cases transported by the rural ambulance service in Bassar, Dankpen, and Keran, which were the active districts in the intervention at the time. This represents 18.8% of the expected cases calculated based on the population-level data (**Table 3**). In the second year of the study period, there were 315 complicated obstetric cases across Bassar, Dankpen, Keran, and Binah, which represents 35.7% of expected cases. In the third year, the IH rural ambulance service transported 66.5% (n = 598) of expected complicated obstetric cases across the four districts (**Figure 2**).

**Table 3:** Coverage of estimated pregnant women with complications served by Integrate Health Ambulance Referral Service in four districts in northern Togo, July 2020 – June 2023

No. of actual complicated obstetric cases, n (% of expected complicated obstetric cases)	Bassar	Dankpen	Keran*	Binah†	Total per year
Year 1, July 2020 – June 2021	42 (19.6)	43 (17.7)	32 (19.4)	N/A†	117 (18.8)
Year 2, July 2021 – June 2022	66 (30.0)	80 (32.3)	111 (50.0)	58 (29.4)	315 (35.7)
Year 3, July 2022 – June 2023	135 (60.1)	73 (29.2)	184 (81.4)	206 (103.9)	598 (66.5)
Total per district	243 (36.9)	196 (26.5)	327 (53.4)	264 (67.3)	1030 (42.8)

*Integrate Health Ambulance Referral Service not available until September 2020.

†Integrate Health Ambulance Referral Service not available until July 2021.

## DISCUSSION

These findings show that a rural ambulance service can improve access to quality EmOC in rural, sub-Saharan Africa with the IH ambulance referral service transferring 66.5% of women with obstetric complications to an established health facility. Although this study was not designed to establish the effect of the ambulance referral service on maternal health outcomes, it is globally recognised that timely access to care for women with obstetric complications is essential for reducing maternal mortality [21]. Understanding the coverage of obstetric complications in northern Togo is a prerequisite to improving maternal health and reducing mortality in the region.

Previous studies have evaluated similar ambulance referral services in India [11], South Sudan [12], Zambia [22], and Burundi [9] with reported EmOC coverage rates at 4.7, 38.6, 68, and 80% respectively. The IH rural ambulance service demonstrated relatively high EmOC coverage rates similar to that in Zambia but lower than the services in Burundi. There are several factors that could explain the broad range in coverage between these programmes including vastly different geography, population size, and pre-existing health care systems. For example, the catchment population served in India was over 1000 times larger than that in northern Togo while the catchment population in Zambia resided in urban rather than rural settings. Additionally, the services in Zambia, India, and Togo were not the only means of transportation to a hospital, which could result in lower coverage rates for these services.

The IH rural ambulance service was most similar to the studies implemented in South Sudan and Burundi in terms of study size, structure of the programme, and focus on rural communities. Researchers in South Sudan and Burundi concluded that the coverage rates of their ambulance programmes were satisfactory, at 38.6 and 80%, respectively, given their demanding settings. Despite the similarities in the services, there were several advantages to the Burundi study that may explain their higher coverage rate. Most importantly, the Burundi study served a district nearly five times smaller, both geographically and in population size, than the Kara region in Togo, with only nine VHCs and one central hospital. Analysing coverage rates at the district rather than regional level in northern Togo demonstrates the potential for higher coverage of EmOC in smaller catchment populations like Binah and Keran, which are of comparable size to the Kabezi district in Burundi and demonstrate the highest coverage rates (>80%) in the third year of the service.

There are several operational strengths of the IH rural ambulance service that likely contributed to its success (**Box 2**). These include multiple adequately resourced referral facilities (i.e. VHC, district hospital) that provide quality EmOC, robust protocols and trained staff coordination to identify women in need of referral, a communication system using cell phones, 24/7 availability of functional, dedicated, and easily accessible ambulances free of charge, and extensive community integration. These features of our ambulance referral service are widely known to collectively contribute to an effective MCH referral system [9,22]; however, the most notable and unique strengths of our ambulance service are tied to our extensive community integration. First, our ambulance service was highly accessible with motorised tricycles stationed at VHCs at all times, compared to other services that stationed and dispatched vehicles from the hospital to the community only when needed. These other services experienced extended referral times and even longer transfer times when ambulances were called far into the community [9]. Previous studies in Sierra-Leone and Malawi have also shown that motorbikes are cost-effective, better equipped to service remote areas with difficult terrain, and most effective when stationed close to the community [14,23]. Another strength of the IH ambulance service is related to the utilization of CHWs through the IPCP initiative. The CHWs monitor at-risk women in the community daily, educate their community on accepted health practices, and serve as a liaison between the community and the health centres. The IPCP initiative established Integrate Health as a trusted and accepted resource in the community prior to or alongside the implementation of its ambulance service. Additionally, CHWs help the service run smoothly by assisting with the early identification of women at risk, facilitating ambulance requests from the community, coordinating with nurses at the VHCs, and ensuring that community members know that the ambulance is both free and available to them.

Box 2 **Operational strengths of the Integrate Health Ambulance Service**

1. Transfer to referral facilities that provide quality emergency obstetric care

2. Robust referral protocols and staff coordination

3. Functional communication system

4. 24/7 availability and free of charge

5. Motorised tricycle design

6. Extensive community integration

a. ambulances stationed within the community

b. community health workers promotion and utilisation

### Limitations

This study has several limitations to address. First, the data collection tool is inherently prone to errors since it involved manually writing responses in an ambulance logbook. The data collectors were ambulance drivers who are non-research staff and were recording data in high-stress, unpredictable, urgent situations. The variables that were recorded did not include socio-demographic characteristics or other key measures such as referral time or clinical outcomes, which limited our ability to conduct a more robust evaluation of the programme. Future evaluations should consider the use of socio-demographic characteristics to identify potential gaps in ambulance service coverage by marital status, education history, ethnicity, language, and wealth, among others. Additionally, comparing women with and without obstetric complications by sociodemographic characteristics may highlight populations at greater risk, which can be targeted for the intervention. The lack of clinical outcomes is a significant limitation of this study because we are unable to determine the effect of the ambulance referral service on maternal morbidity or mortality. We can only report whether women with obstetric complications were transported not whether they received treatment in the health facility, left before receiving treatment, or died while at the facility. These limitations in the data source informed our decision to report descriptive rather than inferential statistics, which lessen the strength of our findings; however, previous evaluations of similar ambulance referral services have successfully demonstrated the effectiveness using descriptive statistics alone [9].

There are also limitations in our definitions of complicated obstetric cases. For women referred under signs of danger, we cannot confirm the exact risk factor for which they were referred. Additionally, we were not able to consider all WHO/UN definitions of complicated obstetric cases such as premature birth of less than 37 weeks, postpartum sepsis, ectopic pregnancy, complications of abortion, intrauterine fetal death with no uterine contractions, and ruptured uterus due to limitations in our data collection tools and compatibility between IH IPCP and WHO/UN definitions [5]. This may result in an underestimation of EmOC cases reported by our study. Furthermore, we may have under- or overestimated EmOC coverage rates due to our reliance on DHIS2 catchment population estimates and the exclusion of the capital district, Kozah. Migration between districts also may distort our district-level estimates due to discrepancies in the eligible and actual population served by the rural ambulance service. The coverage rates may also be underestimated due to variation in geography and culture. For example, some districts are more mountainous and liable to flood, while some remote communities within the districts may not announce pregnancies and, therefore, may not use the ambulance service for fear of reporting complications [5]. Approximately 18 of the 36 months in the study period occurred in the rainy season, which may also significantly affect coverage due to the limited ability of the ambulance to reach the catchment population and limited access to or destruction of existing health care and community infrastructure. For example, there was significant flooding in both 2020 and 2022 that prohibited access to many northern Togolese communities for at least two months each of these years.

Finally, it is possible that we underestimated the EmOC coverage rate in the region if women in the catchment population were using other public ambulances available. The IH service was implemented specifically for rural regions while the public ambulances were stationed in the city centre of each district, operated via car, and charged to the patient according to the distance travelled. It is also possible that we overestimated the EmOC coverage rate if women in the catchment population were previously able to access EmOC but chose to use the IH ambulance service because it was free. Historically poor access to health care in northern Togo and the limitations of the public ambulance service demonstrate that women in the catchment population were likely not accessing EmOC themselves or via the public ambulance service prior to the implementation of the IH ambulance service. Supplemental qualitative data, not reported in this study, supports this assumption with several women in the catchment population testifying that they had no feasible means to get to a health centre before the IH rural ambulance service. The growth in EmOC coverage from year 1 (18.8%) to year 3 (66.5%) also demonstrates the low access to EmOC at the study start, if the IH ambulance service was indeed the only feasible means of transport to higher levels of care.

## CONCLUSIONS

In conclusion, our findings demonstrate that implementing a rural ambulance service in a region with historically high maternal mortality rates results in robust coverage of obstetric complications. Importantly, this coverage steadily improves with each year of implementation, as services are expanded throughout the districts. The IH rural ambulance service can serve as a model for integrating emergency referral services within established health care networks in rural sub-Saharan Africa and robustly evaluating the effectiveness of such a service. Next steps for the IH rural ambulance service include conducting a cost-estimate analysis, evaluating its effectivenesss for the transport of children, improving coordination with VHCs and hospitals to document clinical outcomes and diagnoses after transport, and integrating general lessons learned to improve implementation. Reaching the SDGs set by WHO may be attainable if interventions like these are widely adopted, funded, and adapted to the communities that they serve.
